# Association between core body temperature and mean airway pressure with endotracheal cuff pressure in intubated patients of emergency department

**DOI:** 10.12669/pjms.35.5.886

**Published:** 2019

**Authors:** Zahra Parsian, Farzad Rahmani, Ata Mahmoodpoor, Mahboob Pouraghaei, Maryam Barzegar Jalali, Robab Mehdizadeh Esfanjani, Hassan Soleimanpour

**Affiliations:** 1 Zahra Parsian, Emergency Medicine Research Team, Tabriz University of Medical Sciences, Tabriz, Iran; 2 Farzad Rahmani, Aging Research Institute, Tabriz University of Medical Sciences, Tabriz, Iran; 3 Ata Mahmoodpoor, Anesthesiology Research Team, Tabriz University of Medical Sciences, Tabriz, Iran; 4 Mahboob Pouraghaei, Emergency Medicine Research Team, Tabriz University of Medical Sciences, Tabriz, Iran; 5 Maryam Barzegar Jalali, Students’ Research Committee, Tabriz University of Medical Sciences, Tabriz, Iran; 6 Robab Mehdizadeh Esfanjani, Neurosciences Research Center, Tabriz University of Medical Sciences, Tabriz, Iran; 7 Hassan Soleimanpour, Road Traffic Injury Research Center, Tabriz University of Medical Sciences, Tabriz, Iran

**Keywords:** Body temperature change, Emergency department, Endotracheal intubation

## Abstract

**Background & Objective:**

Endotracheal intubation is routinely performed in the critical situations. In order to prevent microaspiration and tracheal injury endotracheal cuff pressure is important to remain constant between 20 and 30 cmH_2_O. Positive pressure ventilation, duration of intubation, body temperature, and body movements can alter endotracheal cuff pressure. This survey was conducted to evaluate core body temperature and cuff pressure relation with airway pressure simultaneously.

**Methods:**

This was a descriptive analytic study conducted from March 2018 to July 2018 on 150 intubated patients in the emergency department. All were ventilated with SIMV mode and had Ramsi sedation level of 2-3. Mean airway pressure was measured simultaneouly with core body temperature measurement from ventilator monitor. All these parameters were measured 10 times each hour and documented.

**Results:**

There was a statistically meaningful relation between airway pressure and cuff pressure in the primary evaluation (P=0.02, r=0.19), while none of the subsequent evaluations showed meaningful relation (P>0.05). No significant relation was found between cuff pressure and core body temperature in any of the measurements (P>0.05).

**Conclusion:**

The pressure of cuff should be checked repeatedly after intubation because of substantial variation over time. Factors other than core body temperature and airway pressure can influence cuff pressure.

## INTRODUCTION

Endotracheal intubation is routinely performed in the critical situations.^1^ For preventing microaspiration and tracheal injury endotracheal cuff pressure is important to remain constant between 20 and 30 cm H_2_O.^2^ Positive pressure ventilation, duration of intubation, body temperature, and body movements can alter endotracheal cuff pressure.^3^ Beside tremendous benefits of intubation, irreversible complications can ensue from inadequate attention to side effects of procedure. Increased cuff pressure can cause tracheal membrane injury including erosion, inflammation, cartilage rings softening, tracheal dilation, bleeding, infection, tracheal narrowing, on the other hand inadequate cuff inflation, lower than 18cm H_2_O, can culminate in aspiration of upper airway secretions to lungs.^4^ To prevent complications, endotracheal cuff pressure should be measured and documented in several intervals.^5^ As to the best of our knowledge there is no study that evaluates core body temperature and cuff pressure relation with airway pressure simultaneously this survey was conducted.

## METHODS

It was a descriptive analytic study. In a period of six months from March 2018 to July 2018. Ethic committee of Tabriz University of Medical Sciences approved this study with ethic number of IR.TBZMED.REC.1395.303. As no intervention was done on patients, and patients were all intubated no consent was required. All information was kept secret. All intubated patients who were ventilated with SIMV mode in the emergency department of Tabriz Imam Reza hospital, 150 patients, entered the study. All the patients had Ramsi sedation level of 2-3. Core body temperature was measured with American GENIUS2 electronic thermometer through tympanic membrane with holding 3-5 seconds, the Celsius degrees (°C) was used. Mean airway pressure was measured hourly simultaneous with core body temperature measurement from ventilator monitor. The material of endotracheal cuff was Supa (Iran company), and endotracheal cuff pressure was measured by MALLINCKROET GMBH WEST GERMANY with cm H_2_O at the end of expiration while the patients’ Ramsi level of sedation was 2-3 and patients’ heads were aligned with body axes.

All these parameters were measured each hour for 10 times and documented. The measurements were done by trained last year emergency medicine residents who were not involved in the study. The relation of core body temperature and tracheal tube cuff was measured. The relationship between mean airway pressure and cuff pressure was also calculated; two parameters (mean airway pressure and temperature) were subsequently compared with cuff pressure. All parameters were measured in supine position while heads were aligned with body axes, and room temperature was kept 21-24 degree Celsius. Entrance criteria were age above 18 years, being intubated in previous 48 hours, absence of external ear infection or trauma, and tympanic membrane injury. Patients with increased airway pressure or underlying lung diseases, chest trauma, pneumothorax, atelectasis, history of tracheomalasia, pneumonia, asthma, neck and chest tumour or lymphadenopathy were excluded from the study. Collected data were analysed with descriptive and statistical methods using Version 17 SPSS. Results were reported with frequency (percent), Mean, Standard deviation (SD), and mood (Interquartile range). The normality of data was evaluated using Kolmogorov–Smirnov test. The relation between variables was analyzed through linear regression and spearman correlation statistics. For assessing changes of variables over time, repeated measurements were used in this study P-value <0.05 was defined as meaningful.

Demographic findings were reported mean±SD and percent according to the variables. In order to determine relation between core body temperature and mean airway pressure with cuff pressure, linear regression and Pierson correlation coefficient were used. P value less than 0.05 was considered meaningful.

## RESULTS

From patients that contributed in the study 84(56%) were male and 66(44%) were female. Total number of participants were 150. The median of patients’ heart rate was 94/min and the median of blood pressure was 120 mmHg for systole and 80mmHg for diastole. Rapid sequence intubation (RSI) method was used for intubating 133 patients (89.3%) and for 16(10.7%) patients crash method was used. Cuff pressure changes over time (from first measurement to tenth) were statistically meaningful (P<0.0001) (Table-I).

**Table I:** Variation of data during time.

	Cuff Pressure Median(IQR) mmHg	Core Body Temperature Median(IQR) Celsius degree	Airway Pressure Median(IQR) cmH_2_O
1^st^ measurement	100(110-90)	37.6(37.1-36.7)	11.5(14.2-9.6)
2^nd^ measurement	40(40-30)	37.1(37.5-36.8)	11.5(13.5-9.7)
3^rd^ measurement	30(30-30)	37.1(37.5-36.8)	11.5(13.6-9.5)
4^th^ measurement	30(30-25)	37.1(37.5-36.8)	11.6(13.8-9.6)
5^th^ measurement	30(30-25)	37.1(37.4-36.9)	11.5(13.6-9.9)
6^th^ measurement	30(30-25)	37.1(37.4-36.8)	11.5(13.5-9.5)
7^th^ measurement	30(30-25)	37.2(37.4-36.9)	11.7(13.5-9.8)
8^th^ measurement	30(30-25)	37.2(37.4-37)	11.6(13.7-9.6)
9^th^ measurement	30(30-25)	37.2(37.4-36.9)	11.6(13.6-10)
10^th^ measurement	30(30-25)	37.1(37.3-36.9)	11.7(13.6-9.7)
P-value	<0.0001*	0.212	0.698

IQR: Inter Quartile Range.

The variation of core temperature of patients was not statistically meaningful from first measurement to the last one (P=0.212). The median of first measurement’s interquartile range (IQR) was 37.6°C and the l0th measurement median of IQR was 37.1°C (Table-I). The least median of airway pressure was 11.5cm H_2_O and maximum was 11.7cm H_2_O. The airway pressure of patients did not have noticeable changes over time (P=698). There was a statistically meaningful relation between airway pressure and cuff pressure in the primary evaluation (P=0.02, r=0.19), while none of subsequent evaluations showed meaningful relation (P>0.05); which means that neither core body temperature nor airway pressure can predict cuff pressure (Table-II). No significant relation was found between cuff pressure and core body temperature in any of the measurements (P>0.05). The median cuff pressure was 75mmHg in first measurement. This was measured after inflation of cuff with 10 millilitres of room air. When the measured cuff pressure was higher than 25 mmHg it was adjusted to 25 mmHg. The variation of cuff pressure was 15 in 2nd evaluation of cuff pressure. The medians of subsequent variations from 3rd to 10th measurement were all 5 mmHg (Table-III).

**Table II:** Relation between cuff pressure and core body temperature and airway pressure.

	Variables	β	P-value
1^st^ measurement	Core temperature	1.56	0.22
	Airway pressure	-0.89	0.02*
2^nd^ measurement	Core temperature	-0.82	0.46
	Airway pressure	-0.40	0.27
3^rd^ measurement	Core temperature	0.63	0.33
	Airway pressure	-0.20	0.25
4^th^ measurement	Core temperature	-0.94	0.15
	Airway pressure	-0.1	0.54
5^th^ measurement	Core temperature	0.02	0.97
	Airway pressure	0.09	0.45
6^th^ measurement	Core temperature	0.45	0.48
	Airway pressure	-0.001	0.99
7^th^ measurement	Core temperature	-0.57	0.67
	Airway pressure	0.07	0.74
8^th^ measurement	Core temperature	1.52	0.05
	Airway pressure	0.004	0.97
9^th^ measurement	Core temperature	0.83	0.32
	Airway pressure	0.05	0.71
10^th^ measurement	Core temperature	1.05	0.13
	Airway pressure	-0.12	0.26

* meaningful

**Table III:** Changes of cuff pressure during 10 times measurement compared with primary measurement (25mmHg).

	Cuff Pressure Variation Median(IQR)	P-value
1^st^ measurement	75(85-65)	<0.0001
2^nd^ measurement	15(15-5)	<0.0001
3^rd^ measurement	5(5-5)	<0.0001
4^th^ measurement	5(5-0)	<0.0001
5^th^ measurement	5(5-0)	<0.0001
6^th^ measurement	5(5-0)	<0.0001
7^th^ measurement	5(5-0)	<0.0001
8^th^ measurement	5(5-0)	<0.0001
9^th^ measurement	5(5-0)	<0.0001
10^th^ measurement	5(5-0)	<0.0001

## DISCUSSION

The major findings of our study was that endotracheal cuff pressure changes over time in the patients of emergency department. This is contrary to core body temperature and airway pressure both of which did not alter meaningfully. One of the unique features of our study was that the endotracheal cuff pressure was measured for 10 times in the emergency department. Measurements were also simultaneously compared with core body temperature and airway pressure. Previous studies had evaluated cuff pressure in the operating room or ICU. Different studies have studied variables affecting cuff pressure including tracheal tube material, gas composition that cuff is inflated with, position of the patient and body temperature. A survey conducted on patients who underwent neurosurgical procedures, cuff pressure was measured with manometer in different positions. Three positions that cuff pressure was evaluated were head on pins, the end of procedure, before extubation. A meaningful drop of cuff pressures was notable from the primary position (supine) to extubation time. Additionally, a substantial drop in the cuff pressures was discovered in patients who were prone from their primary intubated position (supine) comparing with other three following time points.^6^ Tracheal tube cuff pressure was elevated significantly in patients who were mechanically ventilated with positive pressure.^4^ Tracheal tube cuff pressure was measured in 70 patients, cuff pressure was higher than standard values in 51.4% of patients and lower in 28.6%, and just in 20% it was within normal range. Measured cuff pressure had a meaningful positive relation with temperature over time, according to Saleh and colleagues’ survey.^3^ Another study that evaluated the effect of body temperature on cuff pressure in patients who underwent cardiopulmonary bypass in France showed that decreased body temperature decreases tracheal cuff pressure. In this study 44 patients were studied and pulmonary artery blood temperature was measured compared in two groups of patients, normo thermic which was defined 35-36 °C and hypotermic 28 to 32 °C.^7^

The population of our study was higher and we evaluated airway pressure as well; but we had not measured pulmonary artery temperature. The findings of these two studies were in contrast with our results in which a meaningful relation was not detected. Another study which encompassed 259 intubated patients, 65% of patients had cuff pressure more than standard, which was subsequently decreased to 20%. Following cuff pressure adjustment, there was a meaningful relation between cuff pressure and mean body temperature, but no relation between core body temperature and cuff pressure alteration.^8^ The results of our study is similar to this study as core body temperature was not associated with cuff pressure changes according to our findings; but we did not evaluate mean body temperature which was found as a relating factor in this study. A linear relation was found between maximum airway pressure and cuff pressure in Nasir et al.’s study. In this study cuff under inflation was experienced by more than half of intubated patients and above 70% experienced overinflation, and 44% experienced both. Lack of sedation and period of intubation were related to under inflation, while no cause was identified for over inflation. These findings showed variable cuff pressure in patients who are in ICU.^9^ Tracheal tube material affects cuff pressure according to a study” An in vitro method to measure permeability of gases through a cuff membrane of tracheal tube in conditions relevant to its clinical uses”. This study showed the positives of inflating cuff with combination of nitrous oxide and oxygen compared with air, which increases the cuff pressure.^10^ As cuff pressure increased considerably in the patients, but did not have meaningful relation with core body temperature and tracheal pressure it seems that other factors might contribute to variations in cuff pressure. According to Miure et al existence of nitrogen in cuff affects on cuff pressure. This might be the reason of altercation in cuff pressure in our study; but further studies are required to evaluate the effects of nitrogen on cuff pressure.

### Strength and Limitations of this study

To the best of our knowledge this was the first study that evaluated cuff pressure simultaneously with both airway pressure and core body temperature. As regards limitations of the study; it was conducted in a single hospital which might be an obstacle for concluding a result to be administrable to all patients. The measurements were done for just 10 hours so it can not be an evidence of relation for longer periods of time. The measurements were done by different emergency medicine residents over time, although they were all trained and they were in final year of residency program, this might affect the results. Therefore, we recommend further studies evaluate the parameters by single person.

## CONCLUSION

The pressure of cuff should be checked repeatedly after intubation because of substantial variation over time. Factors other than core body temperature and airway pressure can influence cuff pressure.
